# Exploring the evolving frontiers of sleep deprivation research in the post-COVID-19 era

**DOI:** 10.1097/MD.0000000000041806

**Published:** 2025-03-14

**Authors:** Siddig Ibrahim Abdelwahab, Manal Mohamed Elhassan Taha, Monira I. Aldhahi, Ahmed Ali Jerah, Abdullah Farasani, Saleh Mohammad Abdullah, Ieman A. Aljahdali, Roa Ibrahim, Omar Oraibi, Bassem Oraibi, Hassan Ahmad Alfaifi, Amal Hamdan Alzahrani, Yasir Osman Hassan Babiker

**Affiliations:** a Health Research Centre, Jazan University, Jazan, Saudi Arabia; b Department of Rehabilitation Sciences, College of Health and Rehabilitation Sciences, Princess Nourah bint Abdulrahman University, Riyadh, Saudi Arabia; c Department of Medical Laboratory Technology, Faculty of Applied Medical Sciences, Jazan University, Jazan, Saudi Arabia; d Department of Clinical Laboratory Sciences, Taif University, Taif, Saudi Arabia; e College of Medicine, Tanta University, Tanta, Egypt; f Department of Internal Medicine, Faculty of Medicine, Jazan University, Jazan, Saudi Arabia; g Pharmaceutical Care Administration (Jeddah Second Health Cluster), Ministry of Health, Jeddah, Saudi Arabia; h Department of Pharmacology and Toxicology, College of Pharmacy, King Abdulaziz University, Jeddah, Saudi Arabia; i Department of Surgery, College of Medicine, Jazan University, Jazan, Saudi Arabia.

**Keywords:** COVID-19, scopus, sleep deprivation, thematic mapping, VOSviewer

## Abstract

Sleep deprivation research (SDR) has undergone substantial transformations in the aftermath of the COVID-19 pandemic. This comprehensive study explores the SDR’s evolving trends, hotspots, and conceptual structure in the post-COVID-19 era. Using bibliometric analysis with VOSviewer and Bibliometrix software, we examined the SDR publications from January 2020 to December 2023. Core sources were identified using Bradford law, and a dataset of 4578 English-based data-driven studies was retrieved from the Scopus database. Our analysis revealed a remarkable annual growth rate of 37.92% in SDR from 2020 to 2023, indicating a heightened research focus in this domain. Among prolific authors, Shao emerged as a key contributor, while Sleep ranked prominently as a leading publication venue. The United States and China have emerged as the most impactful countries for advancing SDR research. Uncovering the most cited documents sheds light on various focal points within SDR, including sleep duration, mental health implications, cardiovascular events, the impact of COVID-19 on sleep patterns, dementia, resilience in children, and sleep patterns among preschoolers. Noteworthy keywords that emerged from the analysis encompassed “sleep deprivation,” “depression,” “insomnia,” “COVID-19,” “anxiety,” “fatigue,” and “obesity.” Using a thematic mapping approach, we delineated distinct themes characterized by niche, basic, emerging, and declining patterns. Clusters of SDR research were observed in areas such as “Fatigue,” “Sleep apnea,” “Sleep,” “Sleep deprivation,” “Migraine,” “Caffeine,” and “Recovery.” Furthermore, trending themes in SDR encompassed “Sleep disturbance,” “excessive daytime sleepiness,” and “Gamma-Aminobutyric Acid.” These comprehensive findings provide valuable insights into the current landscape of SDR, illuminating emerging trends, identifying hotspots, and offering directions for future research in this critical field.

## 1. Introduction

Sleep is the main physiological process in human health that serves different purposes. Physical, mental, and emotional well-being requires sufficient sleep duration and quality. Nonetheless, today’s fast-paced society is characterized by a high prevalence of sleep deprivation (SD). SD implies insufficient sleep duration or poor sleep quality due to numerous factors such as work commitments, personal choices of living, and medical conditions.^[1,2]^ To cultivate healthy sleeping habits and create effective interventions, it is important to understand the major impact of SD on human health and performance.^[1,3]^

SD has significant implications for human physical health as it affects several physiological systems. Chronic SD increases the risk of cardiovascular diseases such as hypertension, coronary artery disease, and stroke. Additionally, it may interfere with glucose metabolism, resulting in insulin resistance and an increased predisposition to diabetes mellitus type II. In addition, the immune system suffers from a lack of sleep, leading to a diminished immune response and increased susceptibility to infections.^[3–5]^

Sleep is connected to mental health in both directions; individuals who are deprived of sleep contribute to the onset, worsening, or even mental disturbance itself. The latter includes an increase in mood disorders, such as bipolar disorder or depression, with anxiety disorders becoming worse, which leads to mental illnesses too frequently. Emotion regulation decreases, while emotional reactivity increases under conditions where people lack enough rest at night while experiencing stress resilience.^[2,6]^

SD adversely affects various aspects of human performance. In workplaces, people suffering from insomnia register low productivity levels due to a reduced concentration span, resulting in more frequent errors and accidents. Sleep-deprived people also have weaker cognitive abilities in terms of attention, memory, and decision-making. SD impairs physical performance, which is impaired by SD, including decreased motor coordination, longer reaction times, and poorer sports achievements.^[1,7]^

Studies on the link between COVID-19 and SD have been conducted to underscore the influence of pandemics on sleep patterns and quality. Some factors that contribute to sleep disturbances during this period can be classified as stressors, such as stress, anxiety, and disrupted routines. Various techniques, such as surveys, actigraphy, and polysomnography, have been used to examine this relationship.^[2,4,5,8–10]^ A bibliometric analysis was employed to comprehensively investigate the scientific literature on SD from 2020 to 2023, with a particular focus on emerging trends and themes in the post-COVID-19 era. This research provides invaluable input into how sleep deprivation research (SDR) has evolved over time based on publication trends, influential authors, top journals, research topics, and collaborative networks. This manuscript identifies key research gaps while outlining the potential impacts of the COVID-19 pandemic on sleep-related matters, including sleep patterns. It is a very important information source for researchers who want to understand the overall impact of losing one’s sleep for health care practitioners or even policymakers interested in well-being in general.

## 2. Materials and methods

### 2.1. Search strategy and data retrieval

For bibliographic data, the Scopus database was selected as the primary source of information because it has extensive coverage across all sciences.^[11]^ To capture the appropriate articles, terms related to SD were combined into a search strategy. The Medical Subject Headings (MeSH) database was used to include synonymous terms that would make the search more comprehensive.^[12]^ The MeSH identifier for SD is https://meshb-prev.nlm.nih.gov/record/ui?ui=D012892. The search query included the following: “Sleep Deprivation,” “Inadequate Sleep,” “Insufficient Sleep,” “Insufficient Sleep Syndrome,” “REM Sleep Deprivation,” “Sleep Debt,” “Sleep Fragmentation,” and “Sleep Insufficiency” along with other terms. This study aimed to identify articles that were wide-ranging in covering all relevant themes. Language selection was further narrowed to focus on the original research articles.

### 2.2. Inclusion criteria

**Figure 1.**
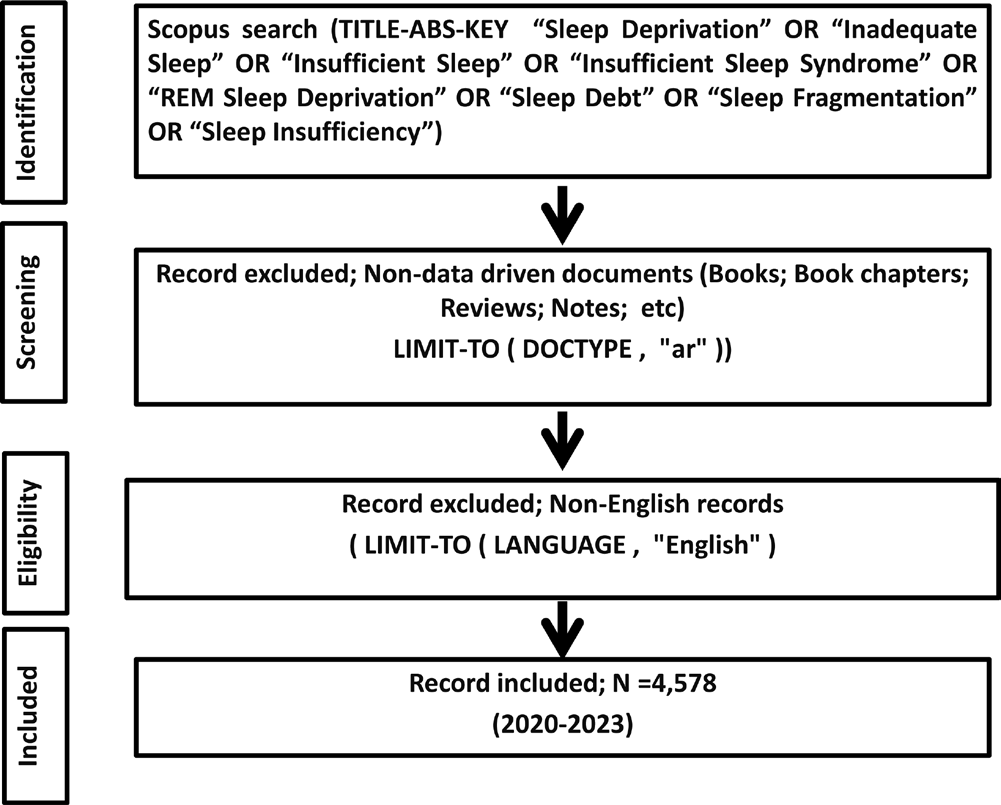
Search strategy.

The study had specific criteria for article inclusion, including indexing in Scopus, being an original research article, and being written in English. As only original research papers were taken into account in this study, it sought to prioritize them while disregarding secondary or review-type publications. In addition, limiting the language to English ensured that there was only 1 dataset throughout the analysis (Fig. 1). The initial search yielded various types of documents. Articles constituted most of the documents, accounting for 70.31% of results. Reviews accounted for 14.47%, whereas book chapters formed 3.53%. Conference papers contributed 2.83%, letters accounted for 2.81%, editorials totaled 2.60%, and notes accounted for 2.21%. Smaller percentages were attributed to brief surveys, errata, books, and conference reviews with 0.78%, 0.23%, 0.16%, and 0.03%, respectively. Thus, in the framework of this research, 4578 data-driven English-based articles were selected and included in the study, which constituted a basis for comprehensive evaluation and thematic mapping analysis carried out in the current study.

### 2.3. Data analysis

Two software tools (VOSviewer version 1.6.20 and Bibliometrix version 4.3.1), both known for their capabilities in bibliometric analysis, were used for the data analysis. The former is a popular tool used to visualize and explore bibliometric networks, whereas Bibliometrix is a good example of a conceptual mapping R package developed into a more comprehensive version.^[13,14]^ Some of these functions supported by these programs include performance analysis, hotspot identification, citation network analysis, collaboration network analysis, among others, as well as other operations like performance analysis within different countries or institutions. By considering connection frequency and network context rather than simple cooccurrence, Total Link Strength (TLS), which is crucial for VOSviewer, goes beyond basic coauthorship detection throughout the entire document content to permit cluster density assessment that ranks item relevance and understanding collaboration patterns as well as citation mapping within scientific landscapes. Ultimately, TLS has become an important instrument for researchers walking through intricate scientific connections, giving them opportunities for making important discoveries.^[13]^

The Bibliometrix software was used to construct a thematic map of the SDR. Key research topics were identified through an analysis of author-assigned keywords. Bibliometrix produces a matrix that shows the links between topics depending on their co-occurrence frequencies. Multidimensional scaling then mapped these topics on the plane, bringing the frequently mentioned keywords together. For the efficient generation of clusters, Bibliometrix further groups keywords into separate themes based on their nearness using automated algorithms that enable clustering with respect to each other. Bibliometrix rates each cluster according to its density score (internal links) and centrality score (relationships to other clusters), which allows it to be assigned into 4 categories: niche themes, motor themes, basic themes, and declining/emerging themes. This final thematic map, plotted by Bibliometrix according to density and centrality scores, helped to identify core topics, trending areas, and research gaps. When combined with bibliographic details, this map provides strategic insights into the landscape of SDRs. This is an efficient way of building a thematic map using bibliometric analyses from Bibliometrix for scientific view mapping and field monitoring purposes.^[14,15]^

### 2.4. Ethical considerations

Not applicable, as this study does not involve human subjects.

## 3. Results

### 3.1. Performance analysis

**Figure 2.**
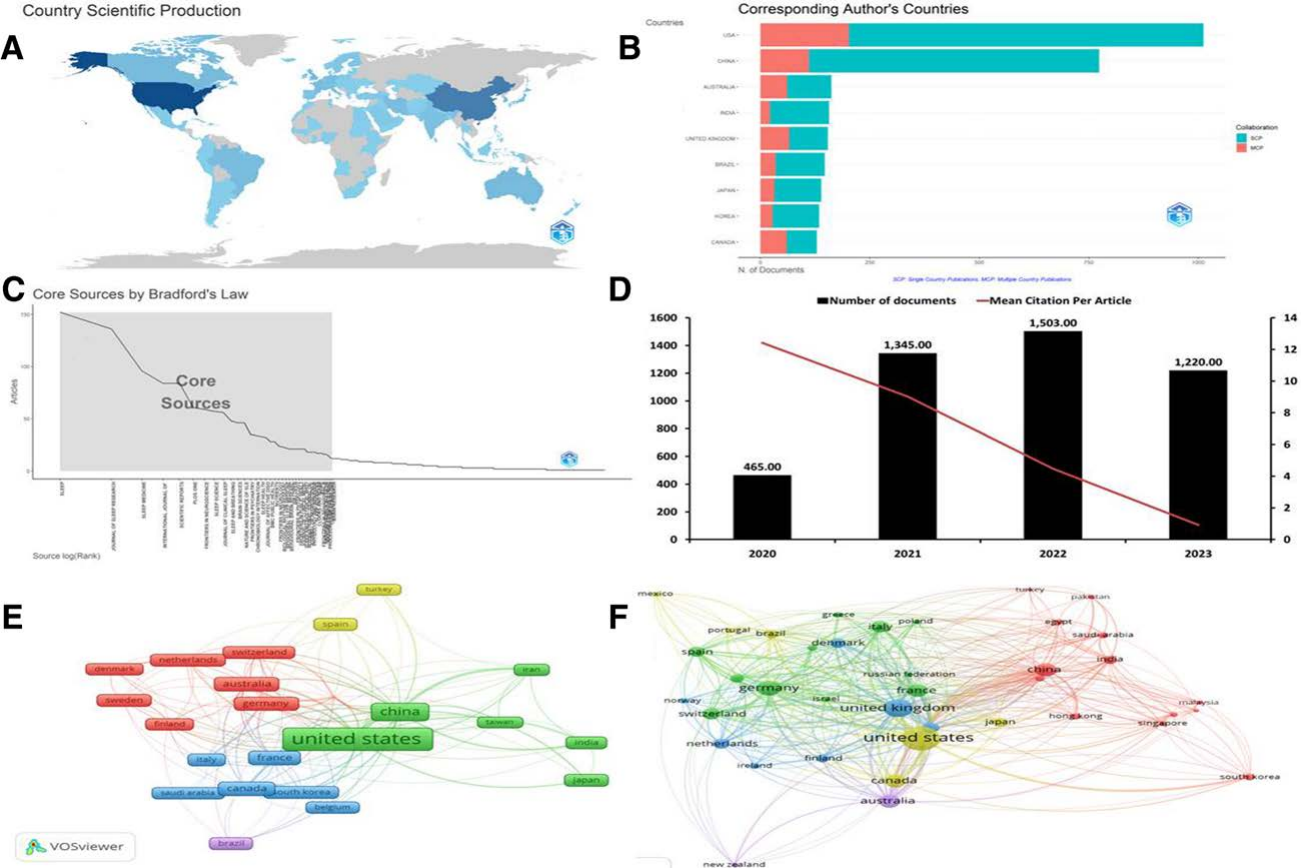
(A) Global production in sleep deprivation research (SDR). Countries with a dark blue color are the most productive. Countries outside the blue category have yet to contribute to research in this area. This figure was generated using the Bibliometrix application and the BibTex data file. (B) An overview of the distribution of multiple-country publications (MCP) and single-country publications (SCP) for different countries. (C) The rankings and zone classifications of the journals according to Bradford law. (D) Annual production and citation of SDR. Y-axes: the number of articles published (left) and citation average (right). X-axis: The year since the first article was published on the subject of this paper. Bars are the number of publication per year. (E) The coauthorship network among countries was analyzed using VOSviewer, representing countries as nodes and collaborations as links. Based on TLS values, the United States emerged as the leading collaborative country in SDR. (F) Highly cited countries. Nodes (TLS) represent the number of total citations. This figure was generated using the VOSviewer application and the CVS data file. CVS = Comma Separated Values.

Out of the dataset, 18,086 authors were found, and 115 wrote single-authored papers. Major contributors to SDR in the post-COVID-19 period include Shao (28 publications), Redline (22 publications), Van Dongen (19 publications), Wright (19 publications), and Peng (18 publications). Additionally, the average number of coauthors per document was 6.6. Harvard Medical School emerged as the most prolific affiliation with 144 publications, followed by Brigham and Women’s Hospital with 77 publications. The Ministry of Education of the People’s Republic of China and the University of Pennsylvania have 73 publications, whereas French National Institute of Health and Medical Research has 65. Figure 2A shows the distribution of research productivity in the SD field across different countries. The United States leads as the most prolific country, accounting for 25.36% of the total publications. China closely followed at 15.30%, while the United Kingdom contributed 4.03% of the publications. Australia, Canada, Japan, Brazil, France, and Germany each had percentages ranging from 3.03% to 3.74%. Collectively, these countries made up a substantial proportion of the research output on SD, totaling 64.32% of the publications.

Figure 2B provides an overview of the distribution of multiple-country publications (MCP) and single-country publications (SCP) for different countries. It also includes the MCP ratio, which represents the proportion of MCP in the total number of publications. Examining the data, India has 135 SCP and 22 MCP with an MCP ratio of 0.14. China has 663 SCP and 111 MCP, resulting in an MCP ratio of 0.143. The United States has 810 SCP and 202 MCP with an MCP ratio of 0.2. Korea has 106 SCP and 28 MCP, yielding an MCP ratio of 0.209. Japan has 107 SCP and 32 MCP, resulting in an MCP ratio of 0.23. Brazil has 112 SCP and 35 MCP, with an MCP ratio of 0.238. Australia has 101 SCP and 61 MCP, with an MCP ratio of 0.377. The United Kingdom has 88 SCP and 66 MCP, yielding an MCP ratio of 0.429. Canada had 69 SCP and 60 MCP, with an MCP ratio of 0.465. These findings offer a comprehensive view of the distribution of MCP and SCP in SDR across various countries and provide insights into their respective MCP ratios.

### 3.2. Bradford law

Bradford law shows a decrease in scatter when one broadens his or her range of references in journals, for example, by using Bradford zones to map out core journals within academic disciplines.^[16]^ In this study, we used Bradford law to identify the core journals on SDR issues, among others. The total number of journals was 1534. The journal ranking and zone classification are shown in Figure 2C. Sleep was ranked first with a frequency of 152, making up the cumulative frequency, and Journal Sleep Research took position 2 with a frequency of 136, cumulating at 288 for zone 1. Sleep medicine ranked third, with a frequency of 96 and a cumulative frequency of 384 in zone 1. The International Journal of Environmental Research and Public Health ranked fourth, with a frequency of 84 and a cumulative frequency of 468 in zone 1. Scientific Reports ranked fifth, with a frequency of 84 and a cumulative frequency of 552 in zone 1. Thirty-nine journals were in zone 1, which absorbed approximately 33% of the SDR. These journals are considered core publications in the SDR based on the application of Bradford law. The largest proportion (38.93%) of these journals fall within the field of medicine. Neuroscience accounted for 17.70%, while Biochemistry, Genetics, and Molecular Biology contributed 9.20% of the publications. Psychology accounted for 6.79% of the research output, while nursing represented 3.54%. Social Sciences and Agricultural and Biological Sciences accounted for 3.12% and 2.94%, respectively.

### 3.3. Annual growth

The commencement of SDR dates back to 1896.^[6]^ However, it is noteworthy that the percentage of original research studies conducted after 2020 was 26% of the total number of original studies since 1896. From 2020 to 2023, SDR has experienced a significant annual growth rate of 37.92% (Fig. 2D). The average age of the research documents during this 4-year period was 2.23 years, indicating a relatively recent output. The average number of citations per document was 5.68, indicating the impact and recognition of the research in the field. Analyzing the mean total citations per article for each year, it was found that in 2020, there was an average of 12.42 citations per article based on 465 articles. However, by 2021, the average decreased by 9.02 citations per article with 1345 articles, followed by a further decrease to 4.47 citations per article, with 1503 articles by 2022. By 2023, the average dropped significantly to 0.92 citations per article, which had dropped significantly to 1220 articles. These findings provide valuable insights into the growth, age, and citation dynamics of SDR from 2020 to 2023.

### 3.4. Mapping of international collaboration and citable SDR

The current dataset presents a fascinating picture of authorship dynamics. While acknowledging the presence of independent researchers through 139 single-authored works, the landscape is dominated by high collaboration, averaging a remarkable 6.6 coauthors per document. Notably, over a quarter of these collaborations transcended national borders, highlighting the global nature of the research in this field. The interplay between independent voices and cross-border partnerships underscores the rich tapestry of knowledge creation in this domain. The coauthorship network analysis depicted in Figure 2E provides deeper insight into the collaborative relationships among countries in the SDR. The figure depicts countries as nodes and collaborations as links. TLS is a significant measure of the amount of collaboration between countries in terms of intensity and frequency. The United States had the highest TLS value and thus emerged as the leading collaborative country, signifying its prominent role in promoting collaboration and participating actively in the SDR on a global basis. However, Figure 2F highlights highly cited countries, with China and the United States being at the top, followed by Canada, Germany, Australia, the United Kingdom, Iran, and France. The figures collectively show that various nations are actively involved in SDR through publication citations and collaboration while focusing on distinct areas of research specialization. They graphically represent research efforts across countries and their impacts on different parts of the world within the SDR domain.

### 3.5. Most cited documents

The most cited documents in the SDR field are listed in Table 1.^[1–5,8–10,17,18]^ These articles covered a range of topics related to the COVID-19 pandemic and its consequences on various aspects of health. They explored the effects of self-quarantine on sleep quality and associated risks, the relationship between sleep duration and dementia in middle and old age, the potential link between sleep loss and gut-related death caused by reactive oxygen species accumulation, the impact of smartphones and social media on youth mental health, the initial psychological effects of the COVID-19 in the Indian community, the association between sleep irregularity and cardiovascular events, the relationship between sleep duration and cognitive decline, the influence of lifestyle factors on high-risk atherosclerosis, vulnerability and resilience of children during the pandemic, and changes in sleep patterns among preschoolers during the COVID-19 outbreak. Overall, these highly cited studies demonstrate the significance of the SDR and its multidimensional impact on human health.

**Table 1:** Most cited documents.

Title	Year	Source	Citation counts
Self-quarantine and weight gain related risk factors during the COVID-19 pandemic	2020	Obesity Research and Clinical Practice	332
Association of sleep duration in middle and old age with incidence of dementia	2021	Nature Communications	205
Sleep loss can cause death through accumulation of reactive oxygen species in the gut	2020	Cell	201
Smartphones, social media use and youth mental health	2020	CMAJ	188
Initial psychological impact of COVID-19 and its correlates in Indian Community: an online (FEEL-COVID) survey	2020	PLoS ONE	179
Sleep irregularity and risk of cardiovascular events: the multi-ethnic study of atherosclerosis	2020	Journal of the American College of Cardiology	174
Association between sleep duration and cognitive decline	2020	JAMA Network Open	159
Lifestyle factors and high-risk atherosclerosis: pathways and mechanisms beyond traditional risk factors	2020	European Journal of Preventive Cardiology	158
Vulnerability and resilience in children during the COVID-19 pandemic	2022	European Child and Adolescent Psychiatry	146
Sleep of preschoolers during the coronavirus disease 2019 (COVID-19) outbreak	2021	Journal of Sleep Research	115

### 3.6. Conceptual structure

#### 3.6.1. Most frequent keywords

**Figure 3.**
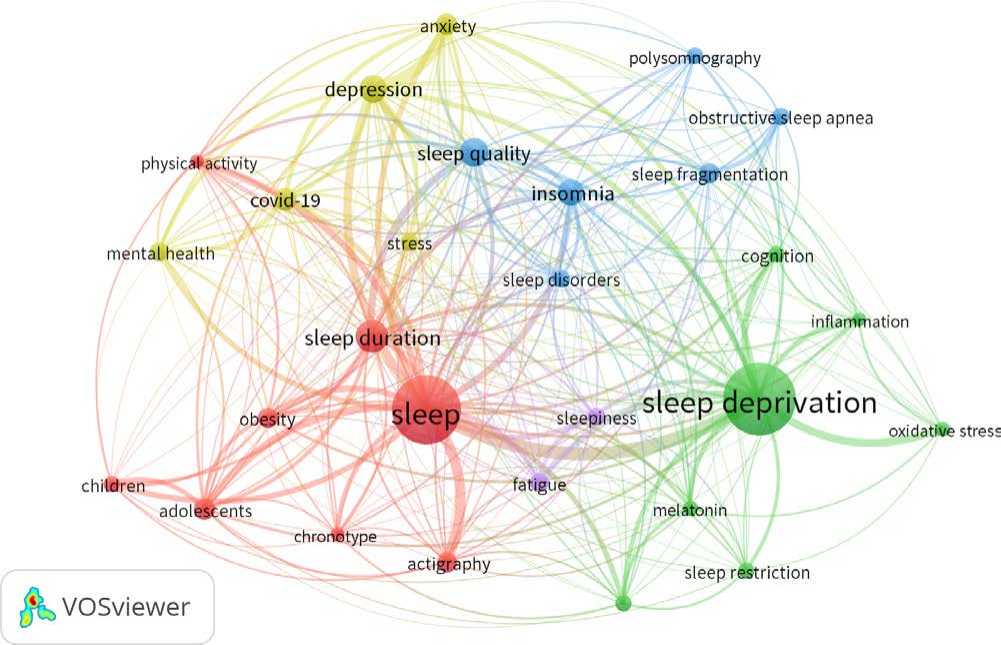
Author’s keyword mapping. The analysis was conducted using VOSviewer, focusing on author keywords with more than 50 occurrences in the dataset.

Building conceptual structures in bibliometric analyses using author keywords is therefore vital. These keywords, which are often obtained from research articles, reflect the principal themes and areas of a specific discipline. When identified, the most frequent keywords enable researchers to understand the knowledge content and error framework of a domain, thereby deepening their understanding of core issues and enhancing focused analysis.^[15]^ A dataset with 2 types of keywords was used: Keywords Plus with 15,785 instances and authors’ keywords with 8218 appearances. New terms were automatically retrieved using DataPlus, and the authors provided their own suggestions. Such words indicate prolific themes found in the dataset, among others. Analysis of the author’s keywords helps researchers comprehend what they should be looking for next. Valuable information on recurring topics and focal points within SDR can be obtained from this list of most common keywords (Fig. 3). VOSviewer was used to perform this analysis based on the author’s keywords having more than 50 occurrences. “Sleep deprivation,” “sleep,” “sleep duration,” and “sleep quality” were top terms that indicated their importance in sleep-related study fields. Other words, such as depression, insomnia, and anxiety, show that there is also a psychological side to SD. In addition, COVID-19 key terms reflected how sleep research has been affected by this pandemic period, and the TLS values attached to each keyword highlight the strength of connections or collaboration associated with them.

### 3.7. Thematic mapping of SDR

**Figure 4.**
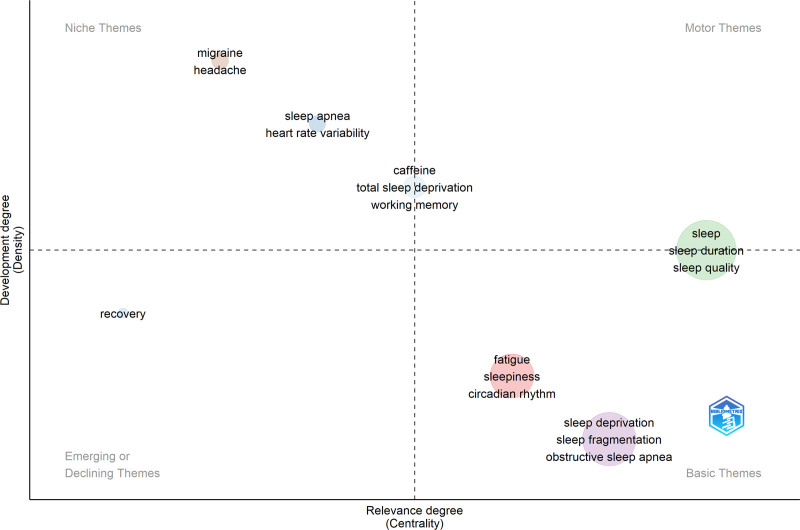
Thematic map of sleep deprivation research based on authors’ keywords. Thematic map is divided into 4 quadrants based on centrality and density, which represent the importance and development of the research topics. This figure was generated using the Bibliometrix application and the BibTex data file.

Figure 4 and Table 2 present a thematic map of the SDR generated using Bibliometrix and BibTex data files. This mapping divides the research themes into 4 quadrants, based on centrality and density. The “basic theme” quadrant features well-established areas like fatigue (centrality: 0.113; density: 3.0*) and SD itself (centrality: 0.120; density: 2.99*). In contrast, the “niche theme” quadrant highlights specialized topics such as sleep apnea (centrality: 0.012; density: 3.78) and migraine (centrality: 0.006; density: 4.35). The “sleep theme” occupies a middle ground, exhibiting moderate centrality and density (0.129 and 3.21*, respectively). Similarly, caffeine fell between the niche and “motor themes” with moderate centrality and density (0.018 and 3.62, respectively). Finally, the “recovery theme” suggests declining importance or development within the field (centrality: 0.002; density: 3.13). This visualization helps researchers understand the prominence and relationships between different areas of SDR, guiding them toward key topics and potential avenues for further investigation.

**Table 2:** Terms of the thematic map.

Cluster	Callon centrality	Callon density	Constructing terms of the cluster	Classification of the cluster
Fatigue	0.11	3.02	Fatigue, sleepiness, circadian rhythm, sleep restriction, melatonin, sleep loss, shift work, exercise, circadian rhythms, pain, alertness, attention, machine learning, vigilance, mood, and cortisol	Basic
Sleep apnea	0.01	3.78	Sleep apnea and heart rate variability	Niche
Sleep	0.13	3.21	Sleep, sleep duration, sleep quality, depression, insomnia, COVID-19, anxiety, adolescents, actigraphy, obesity, mental health, stress, sleep disorders, children, physical activity, chronotype, insufficient sleep, quality of life, epilepsy, epidemiology, prevalence, social jetlag, burnout, child, pregnancy, students, sleep hygiene, risk factors, circadian, sleep health, diet, hypertension, sleep debt, pandemic, public health, delirium, health, and sleep disturbances	Between motor and basic
Sleep deprivation	0.12	2.98	Sleep deprivation, sleep fragmentation, obstructive sleep apnea, cognition, polysomnography, oxidative stress, hippocampus, inflammation, aging, EEG, memory, neuroinflammation, rem sleep, Alzheimer disease, cognitive impairment, gut microbiota, microglia, drosophila, arousal, sleep homeostasis, and memory consolidation	Basic
Migraine	0.01	4.35	Migraine and headache	Niche
Caffeine	0.02	3.62	Caffeine, total sleep deprivation, and working memory	Between motor and niche
Recovery	0.00	3.13	Recovery	Declining

EEG = electroencephalography.

### 3.8. Trending themes

**Figure 5.**
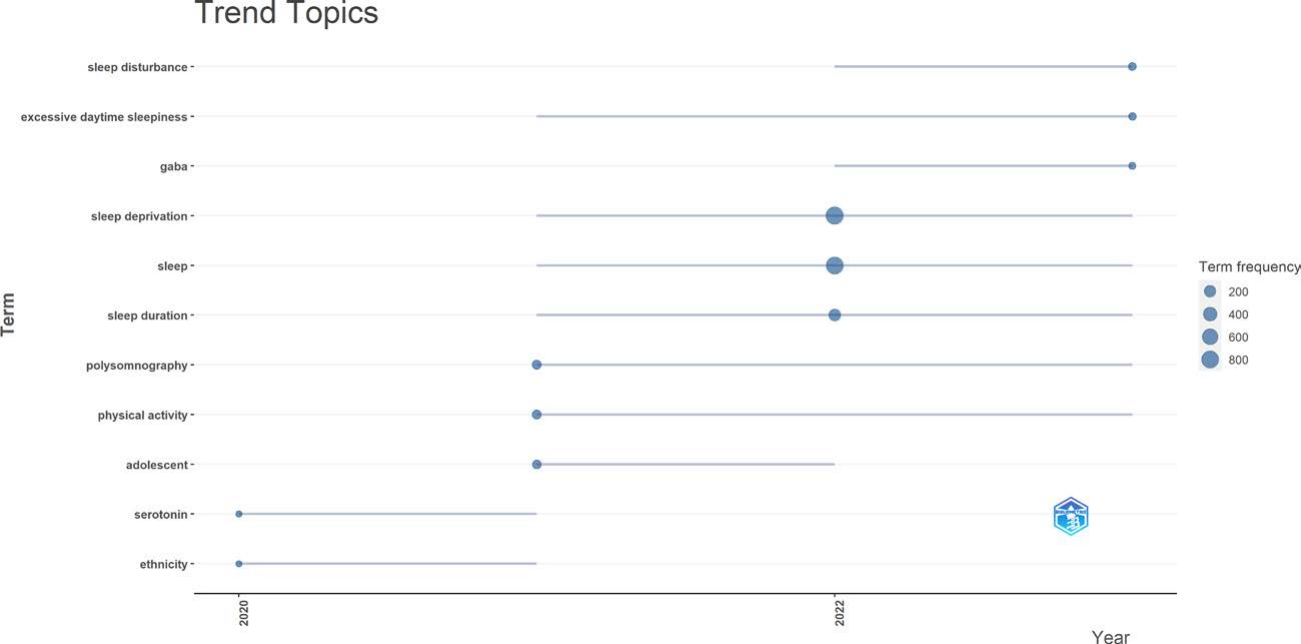
Trending topics.

The trending themes and their dynamics in SDR are shown in Figure 5. “Sleep” reigns supreme with 850 occurrences from 2021 to 2023. “Sleep deprivation” follows closely with 785 occurrences within the same period. “Polysomnography” appears 63 times, mainly in 2021 and 2023. Similarly, “physical activity” will receive a consistent mention (60) from 2021 to 2023. Other prevalent themes include “sleep duration” (223 occurrences), along with “ethnicity,” “serotonin,” “adolescence,” “sleep disturbance,” and “excessive daytime sleepiness,” each with their respective frequencies and temporal distributions shown in the figure. This visualization offers valuable insights into the dominant research topics and their evolving trends in the SDR.

### 3.9. Evolution analysis

**Figure 6.**
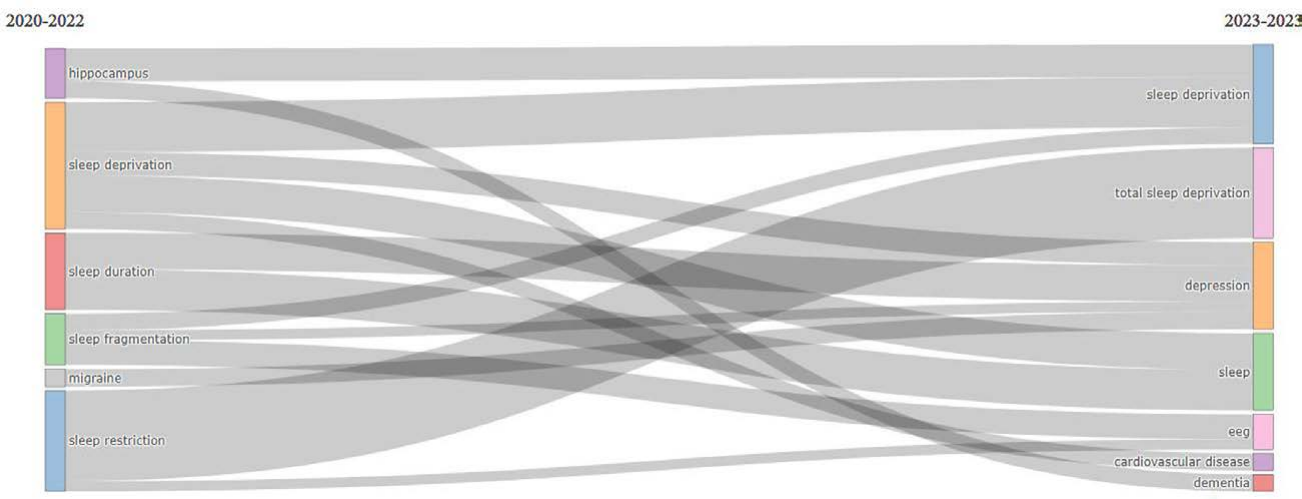
Thematic evolution of the sleep deprivation research. 2022 was a crucial point for the transformation of the main topics. This figure was generated using the Bibliometrix application and the BibTex data file.

Figure 6 presents the thematic evolution of the SDR, delineating the transitions of key themes over a specified timeframe. The initial period from 2020 to 2022 encompasses themes such as hippocampus, migraines, SD, sleep duration, sleep fragmentation, and sleep restriction. However, in the subsequent year, 2023, notable shifts in research focus occurred, leading to the emergence of different thematic trajectories. Specifically, the hippocampal theme gave way to investigations into dementia, while migraines transitioned toward the exploration of depression as a primary concern. SD, although already a prominent theme, continued to feature prominently in the research landscape alongside the emergence of cardiovascular disease and depression as interconnected themes. Furthermore, sleep duration, as a research topic, has expanded to encompass the study of depression and sleep. Sleep fragmentation, previously examined in relation to SD, assumes an additional dimension associated with depression and electroencephalography (EEG). Similarly, sleep restriction extended its purview to include investigations of total SD, in conjunction with depression and EEG analyses. This thematic evolution elucidates the dynamic nature of the SDR, indicating the emergence of novel areas of inquiry and shifting research priorities within the field over a specified temporal trajectory.

## 4. Discussion

Therefore, this article sought to explicate the trending themes in SDR during the post-COVID-19 era. The COVID-19 epidemic has had a significant influence on different aspects of people’s lives, including sleep patterns and quality.^[10,17,18]^ Thus, it is crucial for researchers and health care providers to establish areas that need attention and the implications of SD by comprehending the emerging themes of SDR in these settings. It is important to present an updated understanding of the dynamic nature and effects of this pandemic on sleep patterns and provide insights into targeted interventions and measures aimed at mitigating the effects of SD in the post-COVID-19 period. For this research purpose, with its corresponding benefits, a relevant bibliographic detail was taken from the original, according to previous investigations conducted in this field.^[15,19,20]^

These results indicate that the SDR dates back to 1896. However, 26% of all initial studies were carried out after 2020, implying a low percentage of recent studies in this area alone. This result underlines the necessity for further investigation of the SD. This finding is consistent with previous reports on how the COVID-19 pandemic has affected various aspects of biomedical research.^[21]^ Moreover, between 2020 and 2023, there has been a notable annual growth rate of 37.92%, which suggests a growing interest as well as emphasis on SDR within these years alone. This reflects an increasing recognition as regards why studying so-called SD may be important when viewed eschatologically. In addition, during this time frame, the average age was around 2.23 years, thus denoting recency thereof, indicating that most studies conducted post-2020 are relatively new and also reflect current issues under discussion on SD.^[3,17,18,21]^ In spite of an increase in the number of articles published per annum from 12.42 citations per article in 2020 to 0.92 citations per article in 2023, the average number of citations per article has decreased. These results offer insights into how SDR has grown, aged, and cited between 2020 and 2023. Thus, further studies are needed to determine why there is a fall in average citations.

Because of their complete exploration of the effects of SD on different aspects of health and well-being, these have been the most often quoted works in this field. This has increased our understanding of the association between sleep and various health outcomes, making them highly influential among scientists. For instance, a study investigating the relationship between sleep duration and dementia risk stressed the importance of good sleep for brain function. This finding shows that both short and long sleep durations might increase the likelihood of dementia. The importance of maintaining optimal sleep patterns is highlighted by this discovery; another study addressed the immediate impacts on mental health due to COVID-19, thus providing insights into factors leading to psychological distress as well as resilience. Consequently, we can better understand how crises affect people and, hence, develop appropriate strategies to promote mental wellness.^[3]^ Additionally, studying how smartphones and social media affect youth mental health aims to address concerns over the adverse effects of digital technology on sleeping habits and psychological wellness. Therefore, this study helps us understand that young people should develop the responsible use of digital technologies for better emotional well-being.^[4]^ Moreover, irregularities in sleeping patterns with respect to cardiovascular events provide an indication for establishing regular sleeping habits if we want a healthy heart, according to certain scholars who further warned against erratic sleep schedules, as such are ploys toward developing heart conditions.^[5]^ In addition to the above findings, highly cited articles collectively contribute to SDR fields, among others, by advancing our understanding of the physiological mechanisms, lifestyle factors, and demographic characteristics influencing the relationships between sleep and health outcomes.^[1–3,9,10,17,18]^ These results offer a baseline for future explorations and guide future research efforts aimed at devising interventions or techniques for improving sleep quality while avoiding the negative consequences associated with SD. Consequently, when all these papers were read by most researchers in this field, they also showed their great impact on the science community in shaping our understanding and knowledge about SD as well as its profound effects on health and well-being. Moreover, our investigation builds upon previous studies,^[1–3,9,10,17,18]^ which noted a shift in the research landscape surrounding sleep since the COVID-19 pandemic. This is different from another study that had an overview of trends in sleep research in general, but we take a closer look at 1 area: how research on SD has been evolving during this time period.

Shao is affiliated with Beijing Sport University in China’s School of Psychology, where he has become an eminent author in the field of SDR. This team has conducted several influential studies on this subject. Shao et al investigated the effects of prolonged wakefulness and SD on the brain. They explored alterations in resting-state EEG power spectra and functional connectivity, the impact of different eye states on EEG connectivity, and the restoration of attention processes through sleep recovery after SD. Additionally, research has examined the use of transcranial electrical stimulation to enhance sleep quality restoration and the development of algorithms for the objective assessment of SD-induced mental fatigue using EEG characteristics. These studies have advanced our understanding of the physiological, cognitive, and neurobiological effects of SD.^[22–29]^ Overall, the research by Shao et al has significantly expanded our understanding of the physiological, cognitive, and neural consequences of SD by utilizing various methodologies and gaining recognition within the scientific community for their impactful contributions to the field.

Collaboration allows researchers with different backgrounds and expertise to collaborate.^[15]^ In the context of SDR, experts in fields such as psychology, neuroscience, sleep medicine, and public health can be involved. By combining their knowledge and skills, collaborators can provide a more comprehensive understanding of SD considering its multifaceted nature and diverse implications. The coauthorship network among countries was analyzed using VOSviewer (Fig. 2E), representing countries as nodes and collaborations as links. Based on the TLS values, the United States has emerged as a leading collaborative country in the SDR. Additionally, the average number of coauthors per document was 6.6, indicating a significant level of collaboration among researchers in the SD field during the post-COVID-19 era. A previous study reported that 20% of COVID-19 research papers had >8 listed authors.^[30]^

Figure 2F shows the most highly referenced countries, with China and the United States at the top, followed by Canada, Germany, Australia, the United Kingdom, Iran, and France. These numbers provide a thorough understanding of the worldwide landscape of SDR, highlighting countries that are actively working and earning considerable recognition through citations. China and the United States consistently ranked among the most cited countries in scientific research, including studies on SD. This can be attributed to their robust research infrastructure, significant investment in research funding, active engagement in international collaboration, access to advanced technology and resources, and publication practices in high-impact journals. These factors contribute to the production of high-quality research, which has gained global recognition and attracted citations from the scientific community.^[31]^ However, it is important to acknowledge that other countries (Fig. 2F) also contributed significantly to the SDR, and their citation impact should be considered in the broader context of the field.

The thematic map of SDR includes several clusters: “Fatigue,” “Sleep apnea,” “Sleep,” “Sleep deprivation,” “Migraine,” “Caffeine,” and “Recovery.” These clusters represent key themes and areas of focus in the field. The “Fatigue” cluster explores aspects such as causes, consequences, and interventions related to fatigue. “Sleep apnea” focuses on the sleep disorder characterized by breathing pauses during sleep. The “Sleep” cluster encompasses various aspects of sleep, including architecture, quality, disorders, and their impact on health. “Sleep deprivation” specifically investigates the effects of insufficient sleep on cognition, health, mood, and productivity. The “Migraine” cluster suggests a link between SD and migraines. The “Caffeine” cluster examines caffeine’s impact on sleep and its use as a countermeasure to SD. Finally, the “Recovery” cluster explores strategies for recovering from SD. Together, these clusters formed a comprehensive thematic map covering fatigue, sleep disorders, sleep quality, consequences of SD, and potential interventions.

The theme cluster of “Fatigue” has a Callon Centrality of 0.11 and a Callon Density of 3.02, representing research on fatigue-related topics. The cluster encompassed terms such as sleepiness, circadian rhythm, sleep restriction, melatonin, sleep loss, shift work, exercise, circadian rhythm, pain, alertness, attention, machine learning, vigilance, mood, and cortisol. It is classified as “Basic,” which indicates fundamental research in the field. The cluster demonstrated dense interconnection of terms, indicating thematic coherence and interconnectedness. It covers various dimensions of fatigue, including sleep-related aspects, occupational factors, physical activity, physiological markers, cognitive factors, mood, pain, and the application of machine learning techniques. There is a strong association between fatigue, sleepiness, and detrimental effects on health and performance. Research has shown that individuals who experience fatigue and poor sleep, particularly shift workers, may experience symptoms such as depressed mood, anxiety, and stress. Additionally, fatigue and sleepiness can impair cognitive and physical abilities, similar to the effects of alcohol intoxication, resulting in a notable decline in performance. Moderate levels of fatigue or SD can result in performance decrements equivalent to a blood alcohol concentration of 0.05% to 0.1%, surpassing the legal alcohol limits in certain countries. Moreover, even a loss of 2 hours of sleep has been found to have negative impacts on performance and alertness and increases the risk of errors and accidents. Consequently, the nature of shift work likely contributes to sleepiness, fatigue, and overall poor mental and physical health.^[29,32]^

The theme “Fatigue” also included the keyword “Circadian rhythms.” Foster (2020) emphasized the significant influence of circadian rhythms and the sleep/wake cycle on overall well-being and functioning. This highlights the misconception that humans can disregard biological constraints and operate freely when choosing. In reality, our physiology and behavior are deeply intertwined with a 24-hour internal clock rooted in our evolutionary history. This study explored various themes, including the importance of acknowledging and respecting circadian and sleep systems, the mechanisms underlying their regulation and disruption due to societal pressures and diseases, the interconnectedness of sleep disruption and stress, the frequent cooccurrence of sleep disruption and mental illness, and potential approaches for individuals and employers to mitigate problems associated with working against internal temporal biology. Although some health consequences of sleep disruption can be mitigated, there are always significant negative effects associated with shift work and sleep loss. Consequently, society must confront this issue and determine when the consequences of sleep disruptions are justifiable in the workplace.^[33]^

The theme “Fatigue” also included the keyword “cortisol.” Chronic SD and circadian rhythm disruption significantly affect the release of cortisol, a hormone regulated by the hypothalamic-pituitary-adrenal axis. Both the circadian rhythm and ultradian rhythm of cortisol release are disrupted under conditions of sleep disruption and other stressors. This leads to elevated levels of cortisol and activation of the sympatho-adreno-medullary drive, resulting in the release of adrenaline. Sustained elevation of cortisol and adrenaline levels triggers a stress response that negatively affects various physiological processes, including glucose regulation, immune function, cardiovascular health, digestion, tissue repair, and cognitive function. Laboratory studies have shown that even short-term sleep restriction can replicate the abnormal hypothalamic-pituitary-adrenal secretory activity observed in shift workers and patients with chronic insomnia, emphasizing the importance of adequate sleep for overall health and well-being.^[33,34]^

According to Table 2, the cluster “Sleep apnea” has a Callon Centrality value of 0.01 and a Callon Density value of 3.78. Therefore, the niche was classified as a niche from this analysis. This means that sleep apnea is a type of sleep disorder in broad terms involving sleep disturbance or deprivation of air supply in the body overnight. Consequently, while it is an important area for study, it may not be as widely discussed or investigated as others that relate to sleep. The concept behind the construction of terms within the cluster is based on sleep apnea and heart rate variability (HRV). Sleep apnea is characterized by pauses in breathing or shallow breathing during sleep, which leads to disrupted sleep patterns and inadequate oxygen supply to organs.^[35]^ HRV refers to inter-beat interval oscillations associated with autonomic nervous system activity. Disruption of normal regulation of the autonomic nervous system due to sleep apnea alters HRV. In patients with sleep apnea, there will be reductions in HRV, which are indicators of an increased risk of cardiovascular problems.^[35,36]^ However, further exploration and attention are needed to fully understand and address the complexities of sleep apnea and its related issues.

“Sleep deprivation” theme has a Callon Centrality value of 0.12 and a Callon Density value of 2.98. These values suggest that SD is a relatively basic and essential topic among other general topics regarding sleep disorders. In addition, many terms included within this cluster focused on various impacts related to cognition, memory, and aging.^[1,20]^ However, besides cognitive aspects, there are also some physiological effects of SD that fall into this cluster, including oxidative stress, inflammation, and neuroinflammation.^[2,8,25,26,28]^ Moreover, the inclusion of terms such as gut microbiota, microglia, and Drosophila within the cluster indicated a deeper exploration of the molecular aspects and model organisms involved in studying the effects of SD.^[37]^ The cluster also encompasses references to measuring and diagnosing the effects of SD, such as polysomnography and EEG, which are essential in determining how they affect sleep quality.^[18]^ The all-inclusiveness of this cluster shows that the essential aspect for understanding any other broad field of sleep-related issues, such as health implications, can be SD. Consequently, terms related to sleep fragmentation, obstructive sleep apnea, rapid eye movement sleep, and sleep homeostasis also indicate several other dimensions of SD.^[38]^ Thus, within this particular set of articles, a coherent exploration will lead us through some important facts about cognition and memory aging, among others, caused by SD. By including both consequences and measurement terminology relating to SD, it emphasizes why we should learn and investigate this major factor in order to enhance sleeping standards.

A literature search revealed that during the COVID-19 pandemic, there was a high prevalence of sleep problems, often assessed through self-rating scales or questionnaires.^[39]^ Poor sleep is correlated with anxiety, depression, stress, and posttraumatic stress symptoms, thereby increasing the likelihood of posttraumatic stress disorder. Anxiety can also impair sleep, particularly among health care workers, patients, and those in isolation or quarantine.^[3,17]^ It is important to address sleep problems among health care workers, patients, and the general population. Good sleep and diet are advocated in government strategies, and regulating screen time has been emphasized in schoolchildren during the pandemic. Elderly individuals also need to take care of their sleep habits. Globally, there is a need to promote good sleep practices and mental and emotional health and provide psychological consultations online.^[3,17,21]^ Psychological crisis prevention and addressing misinformation for health care workers are crucial. Adequate rest and sleep should be ensured to prevent burnout and fatigue in health care workers. Nonpharmacological approaches, such as cognitive-behavioral therapy for insomnia and relaxation techniques, can be beneficial.^[40]^ OSA remains an essential characteristic of COVID-19 patients, and guidelines for sleep laboratories and consultations have been issued to prevent the spread of the infection.^[39,41]^ Sleep laboratories should follow recommended infection control procedures, procure personal protective equipment, and prefer telemedicine consultations. Portable home systems and careful disinfection of equipment are recommended for patients with OSA. The restart of sleep labs should consider the local epidemic situation and follow screening guidelines.^[21,39,41]^

The theme of “Migraine” is categorized as a niche concept within the cluster, indicating its specific focus and limited interconnectivity. Although not highly central, it still holds importance and is connected to related terms such as headache, highlighting its relevance in pain and neurological disorders. Migraine and sleep disorders are prevalent chronic conditions that impose a significant burden on individuals and society, leading to substantial socioeconomic consequences.^[42]^ The relationship between migraine and sleep disorders has long been acknowledged by clinicians and supported by epidemiological studies. However, the intricate nature of this association, including its underlying mechanisms and interactions, remains complex and has not yet been fully elucidated. Recent advancements in biochemical and functional imaging research have identified specific structures within the central nervous system and neurotransmitters that play a role in both migraine pathophysiology and regulation of normal sleep architecture. These findings suggest the possibility of dysregulation of common nervous system pathways, potentially contributing to the development of both migraine and sleep disorders. Further exploration of these shared pathways may provide valuable insights into the underlying causes of these conditions and pave the way for targeted therapeutic approaches.^[42–44]^

The current study showed that research on daytime somnolence (excessive daytime sleepiness [EDS]) and the recent trend are happening (Fig. 5). One reason for this may be its commonness, enormous effect on daily life activities and quality, and coexistence with other sleep disorders.^[45,46]^ With public health and safety concerns, researchers are increasingly studying the causes, mechanisms, and possible interventions of EDS. Technological advancements have provided new tools for the objective assessment of sleepiness, leading to more accurate measurements and deeper investigations.^[46,47]^ Therefore, understanding the underlying pathophysiological mechanisms of EDS is important for the development of effective treatment strategies, including pharmacologic options, behavioral therapies, and lifestyle modifications. The mainstay of therapy for EDS due to central disorders of hypersomnolence is pharmacological use of wakefulness-promoting medicines such as modafinil, methylphenidate, amphetamines, or newer agents developed specifically to enhance wakefulness. In addition to pharmacotherapy being used alone, behavioral interventions can also form a useful complement. For cases where EDS is a secondary symptom resulting from other conditions, the primary disorder must be addressed primarily during treatment.^[45–47]^ Researchers advancing knowledge in this area can play a part in improving public safety measures, enhancing productivity levels, and improving overall health outcomes associated with EDS.

However, this bibliometric study had some limitations. Article selection by the study could introduce bias, while reliance on available literature and databases may affect the completeness of the findings. Moreover, the quantitative analysis may have missed qualitative and contextual nuances. Expanding the scope so that non-English publications are also included could increase the range and depth of analysis in this study.

## 5. Conclusions

This study provides several important conclusions and recommendations. First, fatigue has emerged as a fundamental aspect of sleep research, with implications for sleepiness, circadian rhythm, and factors such as pain and mood. Further investigations are warranted to explore its comprehensive impact. Sleep apnea represents a niche area in sleep research, and a deeper exploration of its association with heart rate variability is recommended. The sleep cluster encompasses a wide range of terms, emphasizing the multidimensional nature of sleep and its connections to mental health, obesity, and overall well-being. SD has been highlighted as a significant factor affecting cognition, memory, and neuroinflammation, warranting further investigation into the underlying mechanisms and potential interventions. Migraine has been identified as a specific niche area in sleep research, suggesting a need for further studies on the relationship between migraine and sleep. The effects of caffeine on sleep and cognitive function, particularly in terms of total SD and working memory, require further investigation. Finally, the declining prominence of the “recovery” cluster suggests a potential gap in current research on sleep-related recovery. These conclusions provide valuable insights and highlight specific areas for future investigation in the field of sleep research.

The current research highlights several real-world applications: insights into trends like increased sleep disturbances and mental health impacts can inform targeted public health campaigns; identification of hotspots such as sleep apnea and fatigue can guide health care providers in early screening and personalized treatment strategies; data on sleep pattern disparities can assist policymakers in designing interventions addressing sleep health inequities; findings on SD among children can support school-based programs promoting sleep hygiene and resilience; and trends in sleep research, including studies on Gamma-Aminobutyric Acid and recovery patterns, can drive advancements in sleep-tracking devices and digital health interventions.

## Acknowledgments

The authors would like to thank Princess Nourah bint Abdulrahman University for supporting this project through Princess Nourah bint Abdulrahman University Researchers Supporting Project number (PNURSP2025R286), Princess Nourah bint Abdulrahman University, Riyadh, Saudi Arabia.

## Author contributions

**Conceptualization:** Siddig Ibrahim Abdelwahab, Manal Mohamed Elhassan Taha, Monira Aldhahi, Ahmed Jerah, Abdullah Farasani, Saleh Abdullah, Ieman Aljahdali, Roa Ibrahim, Omar Oraibi, Bassem Oraibi, Hassan Alfaifi, Amal Hamdan Alzahrani.

**Data curation:** Siddig Ibrahim Abdelwahab, Manal Mohamed Elhassan Taha, Ahmed Jerah, Saleh Abdullah, Roa Ibrahim, Hassan Alfaifi, Amal Hamdan Alzahrani.

**Formal analysis:** Siddig Ibrahim Abdelwahab, Manal Mohamed Elhassan Taha, Monira Aldhahi, Saleh Abdullah, Roa Ibrahim, Hassan Alfaifi, Amal Hamdan Alzahrani.

**Funding acquisition:** Siddig Ibrahim Abdelwahab, Manal Mohamed Elhassan Taha, Monira Aldhahi, Abdullah Farasani, Saleh Abdullah, Ieman Aljahdali, Roa Ibrahim, Hassan Alfaifi, Amal Hamdan Alzahrani.

**Investigation:** Siddig Ibrahim Abdelwahab, Manal Mohamed Elhassan Taha, Monira Aldhahi, Saleh Abdullah, Roa Ibrahim, Hassan Alfaifi, Amal Hamdan Alzahrani.

**Methodology:** Siddig Ibrahim Abdelwahab, Manal Mohamed Elhassan Taha, Monira Aldhahi, Saleh Abdullah, Roa Ibrahim, Hassan Alfaifi, Amal Hamdan Alzahrani.

**Project administration:** Siddig Ibrahim Abdelwahab, Manal Mohamed Elhassan Taha, Monira Aldhahi, Saleh Abdullah, Roa Ibrahim, Hassan Alfaifi, Amal Hamdan Alzahrani.

**Resources:** Siddig Ibrahim Abdelwahab, Manal Mohamed Elhassan Taha, Monira Aldhahi, Roa Ibrahim, Omar Oraibi, Bassem Oraibi, Hassan Alfaifi, Amal Hamdan Alzahrani.

**Software:** Siddig Ibrahim Abdelwahab, Manal Mohamed Elhassan Taha, Monira Aldhahi, Roa Ibrahim, Hassan Alfaifi, Amal Hamdan Alzahrani.

**Supervision:** Siddig Ibrahim Abdelwahab, Manal Mohamed Elhassan Taha, Monira Aldhahi, Roa Ibrahim, Hassan Alfaifi, Amal Hamdan Alzahrani.

**Validation:** Siddig Ibrahim Abdelwahab, Manal Mohamed Elhassan Taha, Monira Aldhahi, Roa Ibrahim, Hassan Alfaifi, Amal Hamdan Alzahrani.

**Visualization:** Siddig Ibrahim Abdelwahab, Manal Mohamed Elhassan Taha, Monira Aldhahi, Saleh Abdullah, Roa Ibrahim, Hassan Alfaifi, Amal Hamdan Alzahrani.

**Writing – original draft:** Siddig Ibrahim Abdelwahab, Manal Mohamed Elhassan Taha, Monira Aldhahi, Roa Ibrahim, Hassan Alfaifi, Amal Hamdan Alzahrani.

**Writing – review & editing:** Siddig Ibrahim Abdelwahab, Manal Mohamed Elhassan Taha, Monira Aldhahi, Ahmed Jerah, Abdullah Farasani, Saleh Abdullah, Ieman Aljahdali, Roa Ibrahim, Omar Oraibi, Bassem Oraibi, Hassan Alfaifi, Amal Hamdan Alzahrani, Yasir Babiker.
